# Scrotal hematoma as a sign of adrenal hemorrhage in newborns

**DOI:** 10.1590/S1516-31802011000200011

**Published:** 2011-03-03

**Authors:** Renata Gonçalves, Allan Abuabara, Rubia Fatima Fuzza Abuabara, Claudia Aparecida Feron

**Affiliations:** I MD. Pediatrician and Neonatologist, Dona Helena Hospital, Joinville, Santa Catarina, Brazil.; II DDS. Dentist, Oral and Maxillofacial Radiology Service, Joinville Municipal Authority, Joinville, Santa Catarina, Brazil.

**Keywords:** Hemorrhage, Hematocele, Adrenal glands, Adrenal gland diseases, Scrotum, Hemorragia, Hematocele, Glândulas supra-renais, Doenças das glândulas supra-renais, Escroto

## Abstract

**CONTEXT:**

Bluish discoloration and swelling of the scrotum in newborns can arise from a number of diseases, including torsion of the testes, orchitis, scrotal or testicular edema, hydrocele, inguinal hernia, meconium peritonitis, hematocele, testicular tumor and traumatic hematoma. Forty-two cases of scrotal abnormalities as signs of neonatal adrenal hemorrhage were found in the literature.

**CASE REPORT:**

We present a case of scrotal hematoma due to adrenal hemorrhage in a newborn. Conservative treatment with clinical follow-up was adopted, with complete resolution within 10 days. The possible differential diagnoses are reviewed and discussed.

## Abstract (pt)

**CONTEXTO:**

Edema e coloração azulada do escroto do recém-nascido podem sugerir uma série de doenças, incluindo torção dos testículos, orquite, edema escrotal e testicular, hidrocele, hérnia inguinal, peritonite meconial, hematocele, tumor testicular e hematoma traumático. Quarenta e dois casos de alterações escrotais como sinal de hemorragia da glândula adrenal foram encontrados na literatura.

**RELATO DE CASO:**

Apresentamos um caso de hematoma escrotal devido a hemorragia da glândula adrenal em um recém-nascido. O tratamento adotado foi conservador, com acompanhamento clínico, com resolução completa em 10 dias. Os possíveis diagnósticos diferenciais são revisados e discutidos.

## INTRODUCTION

Bluish discoloration and swelling of the scrotum in newborns can arise from a number of diseases, including hydrocele, torsion of the testes, orchitis, scrotal or testicular edema, inguinal hernia, meconium peritonitis, hematocele, testicular tumor and traumatic hematoma.^1^ Scrotal hematoma can occur secondary to some intra-abdominal diseases, including intraperitoneal or retroperitoneal bleeding.^2^ Neonatal adrenal hemorrhage occurs in about 0.2% of neonates^1^ and scrotal discoloration is an uncommon presentation of neonatal adrenal hemorrhage.^3^ Few cases have been reported in the literature, and the first was by Putnam in 1989.^4^ We report a new case of scrotal hematoma due to adrenal hemorrhage in a newborn.

## CASE REPORT

**Figure 1.**
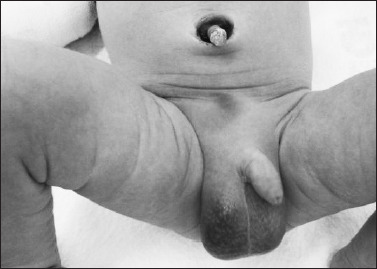
Newborn at the 16^th^ hour of life demonstrating bluish discoloration that extends from the hypogastric region to the right scrotum.

A 2960 g boy was born after gestation of 39 weeks and two days (according to the Capurro method) to a 33-year-old woman by vaginal delivery. The Apgar scores were 2 and 7, respectively, at one and five minutes. The baby was born with the umbilical cord wrapped around his neck. He was hypotonic, bradycardic, pale and without spontaneous breathing. Resuscitation maneuvers were applied. After positive pressure ventilation with a self-inflating bag and mask, he quickly began to breathe spontaneously, and a normal heart rate was restored. Sixteen hours after birth, a bluish discoloration and swelling appeared in the right hemiscrotum and groin (Figure 1). The initial suspicion was acute scrotum.

**Figure 2.**
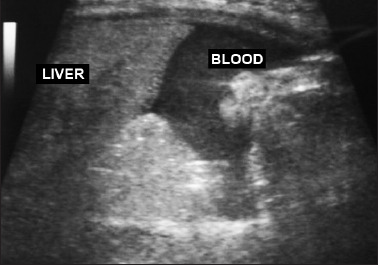
Abdominal ultrasonography showing hemoperitoneum: blood accumulation can be seen near the liver.

**Figure 3.**
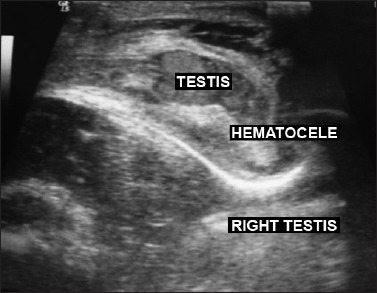
Scrotal ultrasonography showing blood accumulation in the right testis, consistent with hematocele.

**Figure 4.**
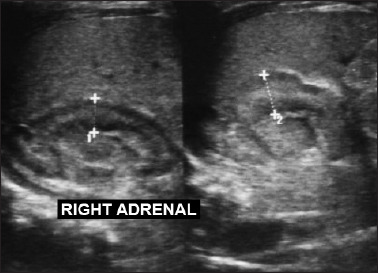
Abdominal ultrasonography showing blood collection around the right adrenal gland, which was enlarged and heterogeneous, consistent with right adrenal hemorrhage.

Ultrasonography showed hemoperitoneum and hematocele in the right hemiscrotum (Figures 2 and 3). The right adrenal gland was enlarged and heterogeneous, consistent with right adrenal hemorrhage (Figure 4). The globular volume decreased from 46.6% to 36.4% over the first twelve hours, thus corroborating the hemorrhagic episode.

Gas analysis showed mild acidosis (pH = 7.19; pO_2_ = 101.9; pCO_2_ = 35; bicarbonate = 14, base excess = −13.9; O_2_ saturation = 96.4%). Correction of acidosis with bicarbonate and conservative treatment were adopted, with complete resolution within 10 days.

## DISCUSSION

In newborns, the adrenal gland is very large and vulnerable to vascular damage.^5^ Neonatal adrenal hemorrhage is more commonly associated with perinatal hypoxia^6^ and difficult or traumatic delivery, or it can be spontaneous.^3^ Ten percent of the cases occur bilaterally.^5^ The hemorrhage is typically contained within the capsule of the adrenal gland, but the capsule may burst, thereby spreading blood in the retroperitoneum or, less frequently, in the peritoneal cavity.^6^ The clinical presentation of adrenal hemorrhage may be asymptomatic, or there may be anemia, persistent jaundice, abdominal mass or, rarely, bluish discoloration and swelling of the scrotum.^7,8^

A search in PubMed (U.S. National Library of Medicine and the National Institutes of Health), Lilacs (Latin American and Caribbean Health Science Literature), SciELO (Scientific Electronic Library Online) and the Cochrane Library databases was conducted using the descriptors [scrotum] and [adrenal gland diseases] and MeSH (Medical Subject Headings), on September 25, 2010. Thirty-one references were found, of which 21 referred to adrenal hemorrhage (Table 1). ^1^–5,^7^–^22^ The main signs and symptoms, risk factors and differential diagnoses linking neonatal adrenal hemorrhage with scrotal abnormalities are shown in Table 2. In the study with the greatest number of cases, Rumińska et al.^7^ presented 13 neonates with adrenal hemorrhage. All the neonates were born at term. The vast majority of the neonates with adrenal hemorrhage (twelve) had risk factors such as birth trauma, intrauterine infection or perinatal asphyxia; there was only one neonate with no risk factors. In our case, the newborn presented the umbilical cord wrapped around his neck, thus resulting in perinatal hypoxia.

**TABLE 1:** Results from a review of the medical databases regarding neonatal adrenal hemorrhage presenting as scrotal abnormalities

Data	Search strategy	Results ^1^–^5^,^7^–^22^	Patients
PubMed	Adrenal Gland Diseases (Mesh) and Scrotum (Mesh)	20 case reports 1 case series^7^	42 neonates
Cochrane Library. SciELO and Lilacs		0	0

MeSH = Medical Subject Headings.

**TABLE 2:** Main signs and symptoms, risk factors and differential diagnoses relating to neonatal adrenal hemorrhage, found in the literature

Signs and symptoms	Jaundice, persistent anemia, discoloration of the scrotum and/or inguinal and perineal areas, scrotal hematoma, acute scrotal swelling, abdominal mass, hypotension or painful swelling of the hemiscrotum and groin
Risk factors	Difficult or traumatic delivery, large birth weight, hypoxia or asphyxia
Differential diagnosis	Torsion of the testes, orchitis, scrotal or testicular edema, hydrocele, inguinal hernia, meconium peritonitis, hematocele, testicular tumor, traumatic hematoma, congenital tumors or cystic neuroblastoma

In newborns with bluish discoloration and hematoma of the scrotum, intra-abdominal disease must be investigated, including intraperitoneal or retroperitoneal bleeding. Scrotal hematoma as a sign of adrenal hemorrhage, as seen in this case, and adrenal neuroblastoma,^19^ are rare but also must be considered. Scrotal and abdominal ultrasonography can provide important information about the patient, and this would seem to be essential, in order to avoid unnecessary surgical exploration.^19^ However, ultrasound rarely distinguishes between adrenal hemorrhage and other causes of suprarenal mass and may suggest a diagnosis of either adrenal hemorrhage or congenital tumors, such as serious cystic neuroblastoma. A postnatal diagnosis of cystic neuroblastoma may be obtained through indirect signs such as 24-hour urine specimen collection to measure vanillylmandelic acid concentration, and by imaging examinations such as computed tomography or magnetic resonance imaging.^5^,^11^

In most of the cases in the literature, the neonatal adrenal hemorrhage was self-limited. Successful conservative treatment with systematic clinical and sonographic follow-up examinations has been reported,^3^ and this approach was also adopted in the present case report.

Drainage using a 23-G needle may be necessary for large adrenal hemorrhage. Surgical exploration may be necessary if the hemorrhage is not controlled or if a hematoma develops into an infected abscess and needs to be drained. Blood or volume replacement may be indicated if the infant has signs of hypovolemic shock. Adrenal insufficiency is rare and transient, and it responds well to steroid replacement therapy.^18^

The outcome is good with complete recovery achieved after a period of between 10 days and six months; residual adrenal calcification has been reported.^3^ Therefore, in newborns with inguinoscrotal swelling and bluish discoloration of the hemiscrotum, it would seem to be essential to perform an ultrasound examination on both the scrotum and the abdomen, in order to discover the possibility of any association with adrenal hemorrhage and avoid unnecessary surgical exploration of the scrotum.
